# LABA/LAMA as First-Line Therapy for COPD: A Summary of the Evidence and Guideline Recommendations

**DOI:** 10.3390/jcm11226623

**Published:** 2022-11-08

**Authors:** Marc Miravitlles, Tomotaka Kawayama, Michael Dreher

**Affiliations:** 1 Pneumology Department, Hospital Universitari Vall d′Hebron, Vall d’Hebron Research Institute (VHIR), Vall d’Hebron Barcelona Hospital Campus, 08035 Barcelona, Spain; 2 Division of Respirology, Neurology, and Rheumatology, Department of Medicine, Kurume University School of Medicine, Kurume 830-0011, Japan; 3 Department of Pneumology and Intensive Care Medicine, University Hospital Aachen, 52074 Aachen, Germany

**Keywords:** COPD, LABA/LAMA, bronchodilator, inhaled corticosteroid

## Abstract

Inhaled bronchodilators (alone or in combination) are the cornerstone of treatment for symptomatic patients with COPD, either as initial/first-line treatment or for second-line/treatment escalation in patients who experience persistent symptoms or exacerbations on monotherapy. The Global Initiative for Chronic Obstructive Lung Disease 2022 report recommends initial pharmacological treatment with a long-acting muscarinic antagonist (LAMA) or a long-acting β_2_-agonist (LABA) as monotherapy for most patients, or dual bronchodilator therapy (LABA/LAMA) in patients with more severe symptoms, regardless of exacerbation history. The recommendations for LABA/LAMA are broader in the American Thoracic Society treatment guidelines, which strongly recommend LABA/LAMA combination therapy over LAMA or LABA monotherapy in patients with COPD and dyspnea or exercise intolerance. However, despite consistent guideline recommendations, real-world prescribing data indicate that LAMA and/or LABA without an inhaled corticosteroid are not the most widely prescribed therapies in COPD. This article reviews global and regional/national guideline recommendations for the use of LABA/LAMA in COPD, examines the evidence for the effectiveness and safety of LABA/LAMA versus other therapies and offers a practical guide for clinicians to help ensure appropriate use of LABA/LAMA therapy.

## 1. Introduction

Chronic obstructive pulmonary disease (COPD) is characterized by airflow limitation and persistent respiratory symptoms (most commonly dyspnea, cough and/or sputum production) and is punctuated by periods of acute worsening, known as exacerbations [1]. Chronic and progressive dyspnea is the most characteristic and debilitating symptom of COPD [1,2]. Activity-related dyspnea has a profound impact on patients’ lives, preventing them from participating in physical activity and often leading to the adoption of a sedentary lifestyle in order to cope with symptoms [3,4,5].

Inhaled bronchodilators (alone or in combination) have become the cornerstone of treatment for symptomatic patients with COPD, either as initial/first-line treatment or for second-line treatment in patients with persistent symptoms or exacerbations despite monotherapy [1,6,7,8]. The Global Initiative for Chronic Obstructive Lung Disease (GOLD) 2022 report recommends initial pharmacological treatment with a long-acting muscarinic antagonist (LAMA) or a long-acting β_2_-agonist (LABA) for the majority of patients, and dual bronchodilator therapy (LABA/LAMA) in patients with more severe symptoms, regardless of exacerbation history [1]. The second-line use of LABA/LAMA is recommended for patients who remain symptomatic despite monotherapy and for those who continue to have exacerbations but are not indicated for add-on therapy with an inhaled corticosteroid (ICS) [1]. A list of currently approved fixed-dose LABA/LAMA combinations is provided in Table 1. The optimal components for LABA/LAMA combination therapy are not considered further in this review but are discussed elsewhere [9,10,11].

National and regional guidelines for COPD are broadly consistent with the GOLD report, recommending single bronchodilator therapy as initial treatment, followed by dual therapy in patients who experience persistent dyspnea and/or exacerbations. However, some guidelines, such as the practical guidance published by the American Thoracic Society (ATS), go further, issuing a strong recommendation for the use of LABA/LAMA over monotherapy in patients with COPD and dyspnea or exercise intolerance [13]. Despite consistent guideline recommendations for the broad use of single/dual bronchodilators as maintenance therapy in patients with COPD, real-world data indicate that bronchodilator therapy without an ICS is not always the most widely prescribed therapy in COPD. Indeed, depending on the country, there may be predominant use of combinations containing ICS (either LABA/ICS or LABA/LAMA/ICS) [14,15,16,17]. This pattern is discordant with global/national guidelines, which consistently reserve their recommendations for ICS-containing combination therapy for a specific subgroup of patients: namely those with a high eosinophil count (≥300 cells/μL), a history of frequent exacerbations (≥2 moderate exacerbations or 1 exacerbation leading to hospitalization in patients with ≥100 eosinophils/μL) or a history of asthma [1,13,18,19,20,21,22,23,24,25]. Possible reasons for this discrepancy include the personal prescribing preferences of physicians and/or their lack of familiarity with treatment guidelines coupled with the late introduction of LAMA to the market relative to LABA/ICS [26,27]. The effectiveness of ICS in treating asthma may be another factor leading to their over-prescription in patients with COPD due to the perceived similarity of the conditions or the co-existence of the two conditions (either real or perceived) [26,27,28]. Physicians may also have an exaggerated focus on exacerbation prevention in COPD compared with the treatment of symptoms that is not aligned with COPD management guidelines [1,29].

In this article, we review global and national guideline recommendations for the use of LABA/LAMA combination therapy in COPD and synthesize the key evidence for the benefits of LABA/LAMA versus (1) monotherapy, (2) LABA/ICS and (3) LABA/LAMA/ICS, drawing on data from systematic reviews, meta-analyses, and individual studies of LABA/LAMA combinations.

## 2. Global and National COPD Treatment Guidelines

Global and national recommendations for the use of LABA/LAMA in the management of COPD are summarized in Table 2 (GOLD, ATS, UK National Institute for Clinical Excellence [NICE], Spanish, German, Japanese, Latin American, Czech, Canadian, Australian and New Zealand guidelines). Consistent across all of these guidelines is the recommendation for the use of a long-acting bronchodilator monotherapy in patients newly diagnosed with COPD who present with mild symptoms (i.e., dyspnea) and infrequent exacerbations (several guidelines such as the Spanish, Czech and Canadian guidelines recommend LAMA specifically in preference to LABA monotherapy). For patients who remain symptomatic with dyspnea despite monotherapy, the majority of guidelines recommend escalation to dual therapy (LABA/LAMA), with the exception of the ATS practical guideline, which recommends LABA/LAMA over LABA or LAMA monotherapy from treatment initiation for patients with dyspnea or exercise intolerance. Similarly, for patients with an eosinophilic phenotype, frequent/severe exacerbations or asthmatic features, the guidelines are consistent in their recommendations for the use of an ICS-containing treatment regimen (LABA/ICS or LABA/LAMA/ICS), though slight variations exist (Table 2). In the following sections, we review the role of LABA/LAMA within the COPD treatment paradigm by describing the evidence for the relative effectiveness of available LABA/LAMA combinations versus long-acting bronchodilator monotherapy, LABA/ICS and LABA/LAMA/ICS.

## 3. Evidence for the Effectiveness and Safety of LABA/LAMA versus Other Therapies

### 3.1. Evidence for the Benefits of LABA/LAMA versus Monotherapies

As shown in Table 3 and Supplementary Table S1, there is a substantial evidence base for the superiority of LABA/LAMA fixed-dose combinations (FDCs) versus LABA or LAMA monotherapy across a wide range of clinical outcomes, including dyspnea, exacerbations, exercise tolerance, health/functional status and health-related quality of life. A Cochrane review of 99 studies including 101,311 patients with moderate-to-severe COPD compared the efficacy and safety of LABA/LAMA FDCs to LABA and LAMA monotherapy. This analysis showed that LABA/LAMA decreased moderate-to-severe exacerbations compared with monotherapy in a high-risk population (≥1 exacerbation in the past 12 months), and there was a general trend towards better symptom control and higher quality of life with LABA/LAMA versus monotherapy. Differences in lung function for LABA/LAMA vs. LABA monotherapy also met the minimal clinically important difference (MCID) in this high-risk population [34]. Other meta-analyses of LABA/LAMA FDCs have reported findings consistent with the Cochrane analysis. In a systematic review and meta-analysis of 19,369 patients with COPD from 10 trials, LABA/LAMA was associated with a lower incidence of all exacerbation events versus LAMA monotherapy in patients with a history of previous exacerbations and those with a longer treatment period (52–64 weeks) [35]. In a meta-analysis of 45,441 patients with COPD from 24 studies, LABA/LAMA was superior to LABA or LAMA monotherapy in reducing the risk of exacerbations and hospitalizations in patients with symptomatic COPD and dyspnea and/or exercise intolerance [36].

Large-scale analyses of specific FDCs have also consistently reported the benefits of dual versus monotherapy. In a pooled analysis of 3699 patients with moderate-to-very-severe airflow limitation and a broad range of COPD symptoms, glycopyrrolate/formoterol (GLY/FOR) was superior to monotherapy in regard to health status, rescue medication use and exacerbation risk. These treatment benefits were more pronounced in patients who had a greater baseline symptom burden, whereas lung function improvements were of a similar magnitude regardless of baseline symptoms, suggesting that dual bronchodilators may have a greater clinical benefit versus monotherapy in symptomatic patients than in patients without symptoms [75]. In a post hoc analysis of the Phase III PINNACLE studies—conducted to assess whether GLY/FOR is appropriate for initial maintenance treatment in COPD compared with LABA and LAMA monocomponents and placebo in maintenance-naïve patients and patients receiving maintenance treatment at screening—results showed that maintenance-naïve patients achieved better lung function with GLY/FOR versus monotherapy and placebo, without an increased safety risk [76].

In a large-scale study of 5162 patients with COPD conducted by Ferguson and colleagues, tiotropium/olodaterol (TIO/OLO) significantly improved lung function (forced expiratory volume in 1 s [FEV_1_] area under the curve from 0–3 s and trough FEV_1_) versus either monotherapy after 52 weeks. This was seen in all GOLD severity groups and in patients both with and without the prior use of LABA or LAMA maintenance therapy, with improvements in lung function mostly seen in patients with less severe disease [77]. In a post hoc analysis of pooled data (1078 patients with COPD naïve to maintenance therapy) from four randomized controlled trials (RCTs) of TIO/OLO versus TIO alone, TIO/OLO demonstrated significant improvements versus TIO alone in trough FEV_1_, SGRQ score and Transition Dyspnea Index (TDI) after 12 weeks [78]. In a post hoc analysis of the TONADO^®^ 1/2 studies, TIO/OLO delayed the time to, and reduced the risk of, clinically important deterioration versus TIO alone in the overall trial population, as well as in patients with a low exacerbation history, patients with GOLD stage 2 COPD (i.e., moderate airflow limitation) and maintenance-naïve patients. These findings suggest that early treatment with TIO/OLO as more effective than TIO alone in reducing the risk of clinically important deterioration in these patient populations [79].

In a pooled analysis of the ARISE, SHINE and SPARK trials, conducted to evaluate the efficacy of indacaterol (IND)/GLY versus LAMA monotherapy (TIO or GLY) in a population of maintenance-naïve patients with moderate-to-very-severe COPD, a greater proportion of patients on IND/GLY achieved minimally clinically important differences in trough FEV_1_, TDI and SGRQ versus monotherapy after 24–26 weeks [80]. These findings are consistent with results from the BLAZE study of 247 patients with moderate-to-severe COPD, in which once-daily IND/GLY was associated with superior improvements in patient-reported dyspnea and lung function after 6 weeks versus placebo (*p* < 0.001) and TIO (*p* = 0.021) [43].

In a pooled analysis of 2 replicate, 52-week studies of 2055 patients conducted to assess whether early treatment with TIO/OLO is more effective than TIO alone in delaying and reducing the risk of clinically important deterioration (CID), TIO/OLO significantly increased the time to, and reduced the risk of, CID versus TIO [81]. Similarly, in an exploratory analysis to assess CID in lung function and health status using an exploratory composite endpoint, dual-bronchodilator therapy with umeclidinium/vilanterol (UMEC/VI) reduced the risk of CID compared with monotherapy or placebo [79]. In the EMAX trial assessing the efficacy of UMEC/VI versus UMEC and salmeterol (SAL) monotherapies in 2431 symptomatic patients with COPD not receiving ICS, UMEC/VI demonstrated sustained improvements in lung function and symptoms and reduced the risk of deterioration/treatment failure (risk of short-term disease deterioration and symptom improvement) versus UMEC or SAL at 24 weeks [82].

Collectively, these results show the benefits of LABA/LAMA versus monotherapy, and this evidence base has already translated into changes in some treatment guidelines, such as those published by ATS (strong recommendation for LABA/LAMA combination therapy over LAMA or LABA monotherapy in patients with COPD and dyspnea or exercise intolerance). The ATS guidelines classify this as a strong recommendation based on moderate-certainty evidence analyzed as part of a systematic review of 24 RCTs extracted from the Embase, Medline and Cochrane libraries [13]. Although most guidelines still recommend a stepwise approach from monotherapy to LABA/LAMA, it is possible that further guideline changes may bring LABA/LAMA forward in the treatment pathway.

### 3.2. Evidence for the Benefits of LABA/LAMA versus LABA/ICS

Several studies have shown the benefits of LABA/LAMA over LABA/ICS in patients with COPD (Table 3 and Supplementary Table S1). A Cochrane review of 11 studies, comprising 9839 participants with mostly moderate-to-severe COPD (without recent exacerbations), compared LABA/LAMA with LABA/ICS. In this analysis, LABA/LAMA was associated with fewer exacerbations, a larger improvement in FEV_1_, a lower risk of pneumonia and more frequent improvement in quality of life, as measured by an increase of ≥4 units in SGRQ total score from baseline [62]. Another Cochrane review conducted on 101,311 participants from 99 studies compared the efficacy and safety of available formulations from four different classes of maintenance therapy (LABA/LAMA, LABA/ICS, LABA, and LAMA) in people with moderate-to-severe COPD. The results of this analysis showed that LABA/LAMA reduced severe exacerbations compared with LABA/ICS (certainty of evidence: moderate) and that LABA/ICS increased the odds of pneumonia compared with LABA/LAMA combination [34].

Studies of specific FDCs have reported similar findings. In the ENERGITO^®^ study, once-daily TIO/OLO provided superior lung function improvements versus twice-daily SAL/fluticasone propionate (FP) (LABA/ICS) after 6 weeks in patients with moderate-to-severe COPD [65]. In a US non-interventional database study assessing 42,953 patients with COPD initiating maintenance therapy with TIO/OLO versus any LABA/ICS combination, TIO/OLO was associated with a lower risk of COPD exacerbations, pneumonia and escalation to triple therapy as well as any one of these events versus LABA/ICS (the combined risk was reduced irrespective of baseline eosinophils or exacerbation history) [64]. In an RCT comparing UMEC/VI to SAL/FP (LABA/ICS), once-daily UMEC/VI over 12 weeks resulted in statistically significant, clinically meaningful improvements in lung function versus twice-daily SAL/FP in patients with moderate-to-severe COPD and infrequent exacerbations, with similar TDI and SGRQ scores in both treatment groups [83]. In a 24-week Phase III trial, treatment with aclidinium/FOR twice daily resulted in a significant increase in trough FEV_1_ versus SAL/FP twice daily [84].

In clinical practice, for the treatment of asthma, the most effective medication available remains low-, medium-, or high-dose ICS; however, for COPD, the guidelines are consistent in their recommendations that ICS treatment be reserved as an add-on therapy for patients who have an eosinophilic phenotype, frequent/severe exacerbations or asthmatic features [1,13,18,20,21,30,85]. If there is uncertainty between a diagnosis of asthma or COPD after careful assessment (current prescription for asthma, history of asthma exacerbations in the years preceding consultation, diagnostic markers), LABA/ICS may be prescribed [86,87].

### 3.3. Comparison of LABA/LAMA versus Triple Therapy

The GOLD report recommends triple therapy for patients with a high eosinophil count (≥300 cells/μL) who remain symptomatic or those with an eosinophil count (≥100 cells/μL) who continue to have exacerbations despite dual therapy with LABA/LAMA or LABA/ICS [1], based partly on the results from two key clinical trials: ETHOS and IMPACT. The ETHOS trial was conducted in a population of 8509 patients with moderate-to-very-severe COPD and a frequent exacerbator phenotype. After 52 weeks, twice-daily triple therapy (budesonide at two different doses plus LABA/LAMA) resulted in a lower rate of moderate or severe COPD exacerbations than dual therapy (GLY/FOR [LABA/LAMA] or budesonide/FOR [LABA/ICS]) [50]. Similarly, in the IMPACT trial, comparison of triple therapy (fluticasone furoate [FF]/UMEC/VI) to dual therapy (FF/VI or UMEC/VI) in 10,355 patients with symptomatic COPD and frequent exacerbations showed that treatment with triple therapy resulted in a significantly lower rate of moderate or severe COPD exacerbations and better lung function and health-related quality of life than dual therapy [51]. In ETHOS and IMPACT, the frequent exacerbator phenotype was defined as ≥1 moderate or severe COPD exacerbations (if FEV_1_ < 50% of predicted normal), or ≥2 moderate or ≥1 severe COPD exacerbations (if FEV_1_ ≥ 50% [ETHOS] or 50–80% [IMPACT] of predicted normal) in the year before screening. In both studies, there was a mortality benefit in the triple therapy arm, but neither study was statistically powered to assess mortality.

The ETHOS and IMPACT studies support the use of triple therapy in patients with a frequent exacerbator and/or eosinophilic phenotype, and subsequent meta-analyses have provided further support for the use of triple therapy in this population (Supplementary Table S1). In a meta-analysis of 16,751 patients with COPD from 14 studies carried out to compare the impact of triple therapy versus LABA/LAMA or LABA monotherapy, results indicated that patients on LABA/LAMA or LABA who still experience exacerbations and have blood eosinophil counts ≥300 cells/µL could benefit from triple therapy by a reduction in exacerbation risk and improvements in trough FEV_1_ [66]. Similar findings were reported in a large meta-analysis by Lee et al. [74]. Triple therapy was the most effective treatment in reducing total exacerbations and mortality, followed by LABA/LAMA. However, this analysis included only a few studies conducted in patients at low exacerbation risk or with a lower symptom burden, and no subgroup analysis was undertaken [74]. In a meta-analysis by Mammen et al. of 14,145 patients from 11 studies, triple therapy was not shown to be superior in reducing exacerbation risk compared with long-acting bronchodilator therapy, except in patients with a history of one or more exacerbations in the previous year (IMPACT accounted for 60% of the weight of the overall analysis) [68]. In a smaller meta-analysis of 632 Japanese patients with symptomatic moderate and severe COPD, triple therapy significantly decreased exacerbations and improved trough FEV_1_ compared with LABA/LAMA therapy [69]. However, Koarai et al. note that this analysis only considered two studies, and the sample size was smaller than the stated optimal information size for each outcome [69]. In another meta-analysis conducted by Koarai et al., triple therapy was superior to LABA/LAMA in terms of the lower incidence of exacerbations and mortality, higher trough FEV_1_ and better quality of life and dyspnea scores [67]. However, this analysis only took into account patients with a history of exacerbations and included a high heterogeneity of studies included for some outcomes (e.g., exacerbations, I^2^ = 78%) [67]. A high level of heterogeneity between studies was also noted in the meta-analysis by Zheng et al., which showed a lower rate of moderate or severe exacerbations of COPD, better lung function and better health-related quality of life for triple therapy compared with dual therapy in patients with advanced COPD [70]. Similarly, although a meta-analysis by Cazzola et al. found a significant reduction in acute exacerbations of COPD with triple therapy versus LABA/LAMA, the heterogeneity of included studies was again high (I^2^ = 98%) [66]. Of note, the reduction in exacerbations was greater in patients with high blood eosinophil counts (≥400 cells/μL) [66]. Finally, analyses from Calzetta et al. showed a superiority of LABA/LAMA/ICS over LABA/LAMA in terms of its efficacy/safety profile; however, the authors noted that three of the four included studies (ETHOS, KRONOS and IMPACT) enrolled some patients with an asthma-like profile, which may potentially bias the findings [88].

Studies comparing triple therapy and LABA/ICS to LABA/LAMA in a broader, more representative population of patients (i.e., infrequent exacerbators) have not replicated the findings of ETHOS, IMPACT or some of the meta-analyses described above in reducing exacerbation rate or mortality [73,89,90,91], supporting the conclusion that the benefits of triple therapy are limited to a high-risk population of frequent exacerbators.

Consistent with recommendations for ICS use in frequent exacerbators, some guidelines recommend ICS withdrawal in patients without exacerbations in the previous year [13,19,92]. Several studies have looked at the efficacy and safety of ICS withdrawal. In the WISDOM trial, patients with severe COPD receiving TIO/SAL/FP had a similar risk of moderate or severe exacerbations whether they continued or discontinued ICS. However, a greater decrease in lung function was observed during the final step of ICS withdrawal particularly in patients with high blood eosinophil counts [93,94]. Results from the SUNSET trial, including 527 patients with COPD on long-term triple therapy without frequent exacerbations, showed that direct de-escalation to IND/GLY led to a small decrease in lung function after 26 weeks but no difference in exacerbation rate [95]. More recently, data from a broad, real-world population of 99,535 patients with COPD including (i) patients meeting the WISDOM trial eligibility criteria (n = 6008); (ii) patients not restricted by the WISDOM trial eligibility criteria (n = 60,645); and (iii) patients who would have been excluded from the WISDOM trial based on their comorbidities (n = 32,882) showed that the rate of FEV_1_ decline was similar between patients on triple therapy and patients who withdrew from ICS regardless of the specific COPD population studied [96].

The increased risk of side effects such as pneumonia associated with ICS use is well documented. In a systematic review of 19 RCTs, exposure to ICS for ≥1 year increased the risk of pneumonia by 41% versus non-ICS-containing treatment regimens. In addition, ICS was associated with an increased risk of tuberculosis and mycobacterial disease and strongly associated with local disorders such as oral candidiasis and dysphonia (an association with the risk of diabetes was only observed at high ICS doses) [97]. In a study by Koarai et al., triple therapy was associated with a significantly higher risk of pneumonia compared with LABA/LAMA (odds ratio 1.52; 95% confidence interval [CI] 1.16–2.00; *p* = 0.003) [67]. Of note, a systematic review of triple therapy versus LABA/LAMA demonstrated that the Japanese population with COPD had double the risk of pneumonia with triple therapy compared with the global population (odds ratio 3.38; 95% CI 1.58–7.22; *p* = 0.002), although these results could not be compared directly [67,69]. In patients with a high risk of pneumonia, including those with a lower body mass index and older age groups, LABA/LAMA may be a safer treatment option than triple therapy [67,68,74]. Results from both the ETHOS and IMPACT trials, comparing triple therapy to dual therapy, showed that the incidence of pneumonia was higher in the treatment groups that received ICS than in those receiving LABA/LAMA [98,99]. Contrary to this cumulative evidence, which strongly indicates that triple therapy should not be recommended as an initial treatment for COPD but rather as a step-up from other combinations therapies, the use of triple therapy as first-line treatment is increasing in primary care. This is highlighted by one Spanish primary care database study in which 34,018 of 197,189 patients (17.2%) with a recorded diagnosis of COPD initiated treatment with triple therapy [100].

## 4. Summary of Recommendations for the Use of LABA/LAMA

**Figure 1:**
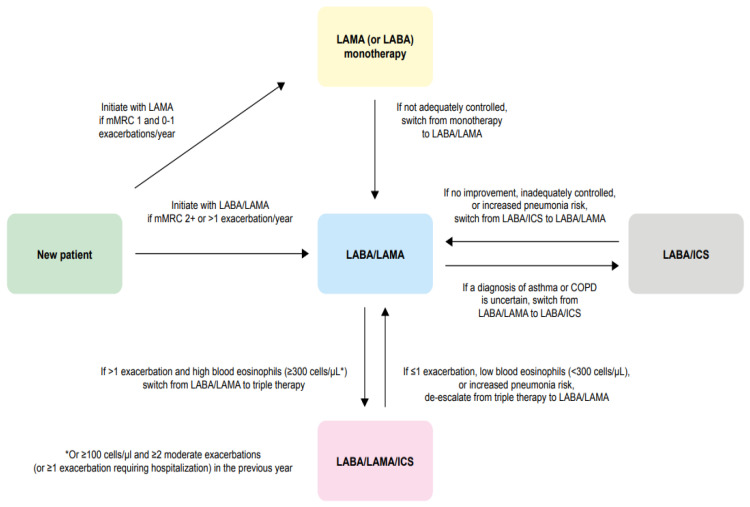
Algorithm to guide physicians in the treatment of COPD. * Or ≥100 cells/µL and ≥2 moderate exacerbations (or ≥1 exacerbation requiring hospitalization) in the previous year. COPD, chronic obstructive pulmonary disease; ICS, inhaled corticosteroid; LABA, long-acting β_2_-agonist; LAMA, long-acting muscarinic antagonist; mMRC, modified Medical Research Council.

Based on global/national guideline recommendations and the available evidence described in this article, we propose a simplified treatment algorithm that we hope will provide physicians with a useful reference guide (Figure 1). We suggest that for patients newly diagnosed with COPD, with a modified Medical Research Council dyspnea scale (mMRC) score of 1 and 0–1 exacerbations/year, mainly LAMA (or alternatively LABA) should be the initial treatment. If the patient has an mMRC score ≥2 or >1 exacerbation/year, the initial treatment should be LABA/LAMA. If a patient’s symptoms are not adequately controlled on monotherapy, treatment should be stepped up to LABA/LAMA combination therapy. If a diagnosis of asthma or COPD is uncertain, patients should start with LABA/ICS or should be switched from LABA/LAMA to LABA/ICS. However, if symptoms show no improvement on LABA/ICS or are inadequately controlled or the patient has an increased pneumonia risk, treatment should be switched to LABA/LAMA (or escalated to LABA/LAMA/ICS depending on the patient’s pneumonia risk). If a patient has had ≤1 exacerbation in the previous year or has an increased pneumonia risk and a low blood eosinophil count (<300 cells/µL), triple therapy should be de-escalated to LABA/LAMA. Conversely, if the patient has had >1 exacerbation in the past year and has a high blood eosinophil count (≥300 cells/µL, or ≥100 cells/µL with ≥2 moderate exacerbations [or ≥1 exacerbation requiring hospitalization] in the previous year), treatment should be escalated from LABA/LAMA to triple therapy.

## 5. Conclusions

Global and national guidelines for the treatment of COPD consistently recommend bronchodilator monotherapy for symptom control at treatment initiation, stepping up to dual bronchodilator therapy (LABA/LAMA) if symptoms persist. However, there is now extensive evidence showing the benefits of LABA/LAMA versus monotherapy, which has translated into changes to some treatment guidelines, such as those published by ATS, which issues a strong recommendation for LABA/LAMA over monotherapy in patients with COPD and dyspnea or exercise intolerance. The evidence we have presented in this review suggests that LABA/LAMA is an appropriate first-line therapy for the majority of patients with COPD who are symptomatic (i.e., breathless) and infrequent exacerbators. Based on the available evidence, ICS-containing therapy (LABA/ICS and triple therapy) should not be used as an initial treatment for COPD but rather as a step-up from bronchodilator therapy if indicated, per global and national guidelines.

## Figures and Tables

**Table 1:** Fixed-dose combinations of LABAs and LAMAs currently approved for COPD treatment [12].

LABA/LAMA	Device	Approved Dose	Frequency of Administration
Tiotropium/olodaterol	Respimat^®^	2.5/2.5 µg *	Once daily
Aclidinium/formoterol	Genuair^®^	340/12 µg ^†^	Twice daily
		400/12 µg ^‡^	Twice daily
Umeclidinium/vilanterol	Ellipta^®^	55/22 µg ^§^	Once daily
		62.5/25 µg ^‖^	Once daily
Glycopyrronium/indacaterol	Breezhaler^®^	85/43 µg ^¶^	Once daily
	Neohaler^®^	27.5/15.6 µg **	Twice daily
Glycopyrronium/formoterol fumarate	Aerosphere^®^	7.2/5 μg ^††^	Twice daily
		9/4.8 μg ^‡‡^	Twice daily

* Approved dose in Europe, the USA, and Japan, two puffs once daily. ^†^ Approved dose in Europe. ^‡^ Approved dose in the USA. ^§^ Approved dose in Europe. ^‖^ Approved dose in the USA and Japan. ^¶^ Approved dose in Europe and Japan. ** Approved dose in the USA. ^††^ Approved dose in Europe and Japan, two puffs twice daily. ^‡‡^ Approved dose in the USA. Dosing information accurate per US, EU, and Japanese prescribing information (accessed on 10 July 2022) and Rhee et al., 2019 [12]. LABA, long-acting β_2_-agonist; LAMA, long-acting muscarinic agent.

**Table 2:** Global consensus on LABA/LAMA in the long-term management of COPD.

Guideline	Dyspnea, Infrequent Exacerbations	Dyspnea, Frequent Exacerbations
GOLD [1]	Initial treatment GOLD A ^1^—bronchodilator GOLD B ^2^—LABA or LAMA Follow-up treatment Escalate to LABA/LAMA if dyspnea not controlled with monotherapy	Initial treatment GOLD C ^3^—LAMA GOLD D ^4^—LAMA or LABA/LAMA (if highly symptomatic) or LABA/ICS (blood eosinophil counts >300 cells/μL) Follow-up treatment Escalate to LABA/LAMA (from monotherapy) if dyspnea/exacerbations not controlled with monotherapy Consider LABA/ICS or LABA/LAMA/ICS if blood eosinophil counts ≥300 cells/μL or ≥100 cells/μL and ≥2 moderate exacerbations/1 hospitalization
ATS [13]	Strong recommendation for LABA/LAMA for patients with dyspnea or exercise intolerance	Conditional recommendation for LABA/LAMA/ICS over LABA/LAMA for dyspnea or exercise intolerance and ≥1 exacerbation/year Conditional recommendation for ICS withdrawal (LABA/LAMA/ICS > LABA/LAMA) if no exacerbations in previous year
NICE [18]	LABA/LAMA for patients who remain breathless or have exacerbations ^5^ For patients with asthmatic features: consider LABA/ICS or LABA/LAMA/ICS	LABA/LAMA for patients who remain breathless or have exacerbations ^5^ For patients with asthmatic features: consider LABA/ICS Consider LABA/LAMA/ICS for those with a severe exacerbation (requiring hospitalization) or 2 moderate exacerbations/year
Spain [19,30]	Low risk ^6^: LAMA as initial treatment, escalated to LABA/LAMA if still symptomatic on monotherapy High risk ^7^: LABA/LAMA as initial treatment for all non-exacerbators	Low risk ^6^: LAMA as initial treatment, escalated to LABA/LAMA if still symptomatic on monotherapy High risk ^7^: Eosinophilic exacerbator (>300 cells/μL): LABA/ICS Non-eosinophilic exacerbator: initial treatment with LABA/LAMA. ICS may be useful in some cases, although its efficacy is inferior
Germany [20]	Initial treatment with a long-acting bronchodilator or LABA/LAMA	Initial treatment with a long-acting bronchodilator or LABA/LAMA ICS should be considered if exacerbations occur despite adequate treatment with long-acting bronchodilators
Japan [21,31]	LABA or LAMA monotherapy to address symptoms in moderate COPD Escalate to LABA/LAMA if symptoms persist despite monotherapy	LABA or LAMA monotherapy to address symptoms in moderate COPD Escalate to LABA/LAMA if symptoms persist despite monotherapy ICS reserved for patients with concomitant asthma
Latin America (ALAT) [32]	A bronchodilator for mild symptoms LABA or LAMA monotherapy to address symptoms in moderate COPD Escalate to LABA/LAMA if persistent dyspnea	High level of evidence for the use of LABA/LAMA in preference to LABA/ICS to improve lung function and frequency of exacerbations, with less risk of pneumonia in patients with moderate–very severe COPD High level of evidence for the use of triple therapy in symptomatic COPD patients with severe–very severe obstruction, risk of exacerbations and blood eosinophil counts ≥300 cells/μL to improve lung function and quality of life and decrease the risk of exacerbations
Czech Republic [23]	Long-acting bronchodilator monotherapy is recommended in patients with lower degree of dyspnea and less impaired lung function, with LAMA preferred over LABA Escalate to LABA/LAMA in patients with persistent dyspnea or decline of lung function despite treatment; de-escalate if serious side effects	LABA/LAMA is recommended for patients with more impaired lung function (FEV_1_ ≤ 50%) and/or who are more symptomatic (mMRC ≥2) ICS should be used in combination with long-acting bronchodilator therapy in patients with frequent exacerbations and higher blood eosinophil count (≥300 cells/μL)
Canada [24]	For patients at low risk of acute exacerbations, LAMA is preferred over LABA Recommends the use of LABA/LAMA for patients experiencing acute exacerbations despite the use of LABA or LAMA monotherapy	Dual therapy (LABA/LAMA or ICS/LABA) is recommended for patients at high risk of acute exacerbations LABA/LAMA/ICS triple therapy is recommended for patients at high risk of exacerbations despite the use of LAMA monotherapy or dual therapy (LABA/LAMA or ICS/LABA)
Australia and New Zealand (TSANZ) [33]	A stepwise approach is recommended regardless of disease severity For short-term symptom relief start with a short-acting bronchodilator (SABA or SAMA) LABA or LAMA if short-acting bronchodilators are insufficient If breathlessness or exacerbations persist with monotherapy, LABA/LAMA is recommended	If breathlessness or exacerbations persist with monotherapy, LABA/LAMA is recommended ICS/LABA is recommended in cases of more severe COPD (FEV_1_ < 50% predicted, with a history of repeated exacerbations), although LABA/LAMA is more beneficial in reducing exacerbations LABA/LAMA/ICS may be an option for patients with moderate-to-severe COPD who require additional treatment.

^1^ GOLD Group A: mMRC 0–1, CAT < 10 and 0 or 1 moderate exacerbation (not leading to hospital admission). ^2^ GOLD Group B: mMRC ≥ 2, CAT ≥ 10 and 0 or 1 moderate exacerbation (not leading to hospital admission). ^3^ GOLD Group C: mMRC 0–1, CAT < 10 and ≥ 2 moderate exacerbations or ≥1 exacerbation leading to hospitalization. ^4^ GOLD Group D: mMRC ≥ 2, CAT ≥ 10 and ≥2 moderate exacerbations or ≥1 exacerbation leading to hospitalization. ^5^ Despite having used or been offered treatment for tobacco dependence if they smoke, optimizing non-pharmacologic management and relevant vaccinations and using a short-acting bronchodilator. ^6^ Low risk (must meet all criteria): FEV_1_ (%) ≥ 50%, 0–1 mMRC, 0–1 exacerbation in the previous year without hospitalization. ^7^ High risk (must meet at least 1 criterion): FEV_1_ (%) < 50%, 2–4 mMRC, 2 or more exacerbations in the previous year or 1 hospitalization. ALAT, Latin American Thoracic Society (Asociación Latinoamericana de Tórax); ATS, American Thoracic Society; CAT, COPD Assessment Test; COPD, chronic obstructive pulmonary disease; FEV_1_, forced expiratory volume in 1 s; GOLD, Global Initiative for Chronic Obstructive Lung Disease; ICS, inhaled corticosteroid; LABA, long-acting β_2_-agonist; LAMA, long-acting muscarinic antagonist; mMRC, modified Medical Research Council; NICE, National Institute for Health and Care Excellence; SABA, short-acting β_2_-agonist; SAMA, short-acting muscarinic antagonist; TSANZ, Thoracic Society of Australia and New Zealand.

**Table 3:** Comparison of LABA/LAMA with monotherapy, LABA/ICS or triple therapy.

LABA/LAMA versus	Lung Function	Dyspnea	Exacerbations	Exercise Tolerance	Health/ Functional Status/ Quality of Life	Pneumonia
LAMA	Rogliani Int J Chron Obstruct Pulmon Dis 2018 ^SR^ [37]	Rogliani Int J Chron Obstruct Pulmon Dis 2018 ^SR^ [37]	Rogliani Int J Chron Obstruct Pulmon Dis 2018 ^SR^ [37]	Rogliani Int J Chron Obstruct Pulmon Dis 2018 ^SR^ [37]	Rogliani Int J Chron Obstruct Pulmon Dis 2018 ^SR^ [37]	Rodrigo Int J Chron Obstruct Pulmon Dis 2017 ^SR/MA^ [38]
	Calzetta Eur Respir Rev 2017 ^MA^ [39]	Calzetta Eur Respir Rev 2017 ^MA^ [39]	Calverley Lancet Respir Med 2018 ^RCT^ [40]	Calzetta Respir Med 2017 ^MA^ [41]	Calzetta Eur Respir Rev 2017 ^MA^ [39]	Oba Cochrane Library 2018 ^SR/MA^ [34]
	Aziz Int J Chron Obstruct Pulmon Dis 2018 ^SR/MA^ [42]	Mahler Eur Respir J 2014 ^RCT^ [43]	Ichinose Int J Chron Obstruct Pulmon Dis 2018 ^RCT^ [44]	O’Donnell Eur Respir J 2017 ^PRCT^ [45]	Ferguson NPJ Prim Care Respir Med 2017 ^PRCT^ [46]	
	Mahler Eur Respir J 2014 ^RCT^ [43]	Ferguson NPJ Prim Care Respir Med 2017 ^PRCT^ [46]	Wedzicha Adv Ther 2020 ^PRCT^ [47]	Minakata Int J Chron Obstruct Pulmon Dis 2019 ^PRCT^ [48]	Martinez Int J Chron Obstruct Pulmon Dis 2019 ^PRCT^ [49]	
	Martinez Int J Chron Obstruct Pulmon Dis 2019 ^PRCT^ [49]	Martinez Int J Chron Obstruct Pulmon Dis 2019 ^PRCT^ [49]	Chen Ther Adv Respir Dis 2020 ^SR/MA^ [35]	Ichinose Int J Chron Obstruct Pulmon Dis 2018 ^RCT^ [50]	Price Int J Chron Obstruct Pulmon Dis 2017 ^SR^ [51]	
	Price Int J Chron Obstruct Pulmon Dis 2017 ^SR^ [51]	Price Int J Chron Obstruct Pulmon Dis 2017 ^SR^ [51]	Mammen et al. Ann Am Thorac Soc 2020 a^SR/MA^ [36]	Maltais Adv Ther 2021 ^MA/PRCT^ [52]	Buhl Eur Respir J 2015 ^PRCT^ [53]	
	Buhl Eur Respir J 2015 ^PRCT^ [53]	O’Donnell Eur Respir J 2017 ^PRCT^ [45]		Takahashi Int J Chron Obstruct Pulmon Dis 2020 ^RCT^ [54]	Singh Respir Med 2015 ^PRCT^ [55]	
	Singh Respir Med 2015 ^PRCT^ [55]	Miravitlles Respir Res 2017 ^SR/MA^ [56]			Labor Respiration 2018 ^SR^ [57]	
	Beeh Pulm Pharmacol Ther 2015 ^RCT^ [58]	Rodrigo Int J Chron Obstruct Pulmon Dis 2017 ^SR/MA^ [38]			Miravitlles Respir Res 2017 ^SR/MA^ [56]	
	Maltais Adv Ther 2019 ^RCT^ [59]	Takahashi Int J Chron Obstruct Pulmon Dis 2020 ^RCT^ [54]			Rodrigo Int J Chron Obstruct Pulmon Dis 2017 ^SR/MA^ [38]	
	Miravitlles Respir Res 2017 ^SR/MA^ [56]	Calzetta Chest 2016 ^SR/MA^ [60]			Calzetta Chest 2016 ^SR/MA^ [60]	
	Rodrigo Int J Chron Obstruct Pulmon Dis 2017 ^SR/MA^ [38]	Mammen et al. Ann Am Thorac Soc 2020 a^SR/MA^ [36]			Mammen et al. Ann Am Thorac Soc 2020 a^SR/MA^ [36]	
	Calzetta Chest 2016 ^SR/MA^ [60]	Maltais Eur Respir J 2019 ^RCT^ [61]				
	O’Donnell Eur Resp J 2017 ^PRCT^ [45]					
	Ichinose Int J Chron Obstruct Pulmon Dis 2018 ^RCT2^ [50]					
	Maltais Adv Ther 2021 ^MA/PRCT^ [52]					
	Takahashi Int J Chron Obstruct Pulmon Dis 2020 ^RCT^ [54]					
LABA	Rogliani Int J Chron Obstruct Pulmon Dis 2018 ^SR^ [37]	Rogliani Int J Chron Obstruct Pulmon Dis 2018 ^SR^ [37]	Rogliani Int J Chron Obstruct Pulmon Dis 2018 ^SR^ [37]	Rogliani Int J Chron Obstruct Pulmon Dis 2018 ^SR^ [37]	Rogliani Int J Chron Obstruct Pulmon Dis 2018 ^SR^ [37]	Oba Cochrane Library 2018 ^SR/MA^ [34]
	Calzetta Eur Respir Rev 2017 ^MA^ [39]	Calzetta Eur Respir Rev 2017 ^MA^ [39]	Mammen et al. Ann Am Thorac Soc 2020 a^SR/MA^ [36]	O’Donnell Eur Respir J 2017 ^PRCT^ [45]	Calzetta Eur Respir Rev 2017 ^MA^ [39]	
	Price Int J Chron Obstruct Pulmon Dis 2017 ^SR^ [51]	Ferguson NPJ Prim Care Respir Med 2017 ^PRCT^ [46]			Ferguson NPJ Prim Care Respir Med 2017 ^PRCT^ [46]	
	Beeh Pulm Pharmacol Ther 2015 ^RCT^ [58]	Price Int J Chron Obstruct Pulmon Dis 2017 ^SR^ [51]			Price Int J Chron Obstruct Pulmon Dis 2017 ^SR^ [51]	
	Miravitlles Respir Res 2017 ^SR/MA^ [56]	Miravitlles Respir Res 2017 ^SR/MA^ [56]			Miravitlles Respir Res 2017 ^SR/MA^ [56]	
	Calzetta Chest 2016 ^SR/MA^ [60]	Calzetta Chest 2016 ^SR/MA^ [60]			Calzetta Chest 2016 ^SR/MA^ [60]	
	O’Donnell Eur Respir J 2017 ^PRCT^ [45]	O’Donnell Eur Respir J 2017 ^PRCT^ [45]			Labor Respiration 2018 ^SR^ [57]	
		Mammen et al. Ann Am Thorac Soc 2020 a^SR/MA^ [36]			Mammen et al. Ann Am Thorac Soc 2020 a^SR/MA^ [36]	
LABA/ICS	Horita Cochrane Database Syst Rev 2017 ^CR^ [62]	Rogliani Int J Chron Obstruct Pulmon Dis 2018 ^SR^ [37]	Horita Cochrane Database Syst Rev 2017 ^CR^ [62]		Horita Cochrane Database Syst Rev 2017 ^CR^ [62]	Suissa Chest 2019 ^RWS^ [63]
	Rogliani Int J Chron Obstruct Pulmon Dis 2018 ^SR^ [37]	Miravitlles Respir Res 2017 ^SR/MA^ [56]	Rogliani Int J Chron Obstruct Pulmon Dis 2018 ^SR^ [37]		Rogliani Int J Chron Obstruct Pulmon Dis 2018 ^SR^ [37]	Quint Adv Ther 2021 ^RWS^ [64]
	Aziz Int J Chron Obstruct Pulmon Dis 2018 ^SR/MA^ [42]	Rodrigo Int J Chron Obstruct Pulmon Dis 2017 ^SR/MA^ [38]	Rodrigo Int J Chron Obstruct Pulmon Dis 2017 ^SR/MA^ [38]		Miravitlles Respir Res 2017 ^SR/MA^ [56]	Horita Cochrane Database Syst Rev 2017 ^CR^ [62]
	Beeh Int J Chron Obstruct Pulmon Dis 2016 ^RCT^ [65]		Quint Adv Ther 2021 ^RWS^ [64]		Rodrigo Int J Chron Obstruct Pulmon Dis 2017 ^SR/MA^ [38]	Rodrigo Int J Chron Obstruct Pulmon Dis 2017 ^SR/MA^ [38]
	Miravitlles Respir Res 2017 ^SR/MA^ [56]		Suissa Chest 2019 ^RWS^ [63]			
	Rodrigo Int J Chron Obstruct Pulmon Dis 2017 ^SR/MA^ [38]					
Triple therapy	Cazzola Eur Respir J 2018 ^SR/MA^ [66]	Koarai Respir Res 2021 ^SR/MA^ [67]	Cazzola Eur Respir J 2018 ^SR/MA^ [66]		Koarai Respir Res 2021 ^SR/MA^ [67]	Mammen Annals ATS 2020 b^SR/MA^ [68]
	Koarai Respir Res 2021 ^SR/MA^ [67]	Mammen Annals ATS 2020 b^SR/MA^ [68]	Koarai Respir Res 2021 ^SR/MA^ [67]		Koarai Respir Investig 2022 ^SR/MA^ [69]	Zheng The BMJ 2018 ^SR/MA^ [70]
	Koarai Respir Investig 2022 ^SR/MA^ [69]		Cabrera Ann Epidemiol 2022 ^RWS^ [71]		Zheng The BMJ 2018 ^SR/MA^ [70]	Quint Expert Rev Respir Med 2022 ^RWS^ [72]
	Zheng The BMJ 2018 ^SR/MA^ [70]		Quint Expert Rev Respir Med 2022 ^RWS^ [72]			Koarai Respir Res 2021 ^SR/MA^ [67]
			Suissa Chest 2020 ^RWS^ [73]			Suissa Chest 2020 ^RWS^ [73]
			Koarai Respir Investig 2022 ^SR/MA^ [69]			Cazzola Eur Respir J 2018 ^SR/MA^ [66]
			Lee PLOS Med 2019 ^SR/MA^ [74]			Koarai Respir Investig 2022 ^SR/MA^ [69]
			Mammen Annals ATS 2020 b^SR/MA^ [68]			Lee PLOS Med 2019 ^SR/MA^ [74]
			Zheng The BMJ 2018 ^SR/MA^ [70]			

Color code: LABA/LAMA superior; LABA/LAMA equal; LABA/LAMA inferior. Although the prespecified crude analysis produced a rate ratio of 0.93 (*p*-value > 0.01, not significant) comparing LABA/LAMA to LAMA alone, a sensitivity analysis adjusted for the baseline rate of exacerbations and other factors produced a rate ratio of 0.89 (*p*-value 0.001, significant). CR, Cochrane review; ICS, inhaled corticosteroid; LABA, long-acting β_2_-agonist; LAMA, long-acting muscarinic antagonist; MA, meta-analysis; PRCT, pooled or post hoc analysis of randomized clinical trials; RCT, randomized clinical trial; RWS, real-world study; SR, systematic review.

## Data Availability

Not applicable.

## Supplementary Materials

The following supporting information can be downloaded at: https://www.mdpi.com/article/10.3390/jcm11226623/s1, Table S1: Meta-analyses comparing LABA/LAMA with monotherapy, LABA/ICS or triple therapy.
