# Supplementary material for: LABA/LAMA as First-Line Therapy for COPD: A Summary of the Evidence and Guideline Recommendations
Source: J Clin Med. 2022 Nov 8;11(22):6623. doi: 10.3390/jcm11226623 (PMC9692772; doi:10.3390/jcm11226623)
Supplement: Supplementary file 1 [file jcm-11-06623-s001.zip › jcm-1979164-supplementary.pdf]

Supplementary

Table S1. Meta-analyses comparing LABA/LAMA with monotherapy, LABA/ICS or triple therapy.

Reference	Comparison	Outcome	Findings
Oba Cochrane Library 2018 [34]	LABA/LAMA vs LAMA, LABA and LABA/ICS	Moderate-to-severe exacerbations	Vs LAMA: OR 0.96 (95% CI 0.75–1.23; *p*=0.77; I^2=52%)
			Vs LABA: OR 0.77 (95% CI 0.62–0.97; *p*=0.02; I^2=0%)
			Vs LABA/ICS: OR 0.86 (95% CI 0.74–1.00; P=0.05; I^2=10%)
		SGRQ (change from BL at 12 months)	Vs LAMA: MD –1.15 (95% CI –2.24 to –0.06; P=0.04; I^2=34%)
			Vs LABA: MD –0.69 (95% CI –1.64 to 0.25; P=0.15; I^2=0%)
			Vs LABA/ICS: MD –1.2 (95% CI –2.34 to –0.06; P=0.04; I^2=N/A)
		TDI (change from BL at 12 months vs LAMA and LABA and 6 months vs LABA/ICS)	Vs LAMA: MD 0.21 (95% CI 0.1–0.33; P=0; I^2=0%)
			Vs LABA: MD 0.42 (95% CI 0.06–0.77; P=0.02; I^2=76%)
			Vs LABA/ICS: MD 0.13 (95% CI –0.24 to 0.51; P=0.48; I^2=0%)
		FEV_1 (change from BL at 12 months, L)	Vs LAMA: MD 0.06 (95% CI 0.04–0.08; P<0.0001; I^2=65%)
			Vs LABA: MD 0.07 (95% CI 0.06–0.09; P<0.0001; I^2=45%)
			Vs LABA/ICS: MD 0.06 (95% CI 0.04–0.08; P<0.0001; I^2=N/A)
		Mortality	Vs LAMA: OR 1.01 (95% CI 0.75–1.36; P=0.96; I^2=0%)

			Vs LABA: OR 1.19 (95% CI 0.68–2.09; P=0.54; I^2=0%)
			Vs LABA/ICS: OR 1.01 (95% CI 0.61–1.68; P=0.96; I^2=0%)
		Pneumonia	Vs LAMA: OR 1.13 (95% CI 0.83–1.53; P=0.43; I^2=0%)
			Vs LABA: OR 1.54 (95% CI 0.95–2.49; P=0.08; I^2=0%)
			Vs LABA/ICS: OR 0.57 (95% CI 0.39–0.84; P=0; I^2=0%)
Chen Ther Adv Respir Dis 2020 [35]	LABA/LAMA vs LAMA (RCTs with treatment duration of 24–64 weeks)	Time to first exacerbation	HR 0.96 (95% CI 0.79–1.18; P=0.71, I^2=46%)
		Moderate-to-severe exacerbations	Risk ratio 0.96 (95% CI 0.90–1.03; P=0.28, I^2=16%)
		Severe exacerbations	Risk ratio 0.92 (95% CI 0.81–1.03, P=0.15, I^2=0%)
		All exacerbations	Risk ratio 0.92 (95% CI 0.86–1.00; P=0.04, I^2=0%)
Mammen Ann Am Thorac Soc 2020a [36]	LABA/LAMA vs LABA or LAMA (study duration of 2–62 weeks)	Dyspnea	SMD 0.10 (95% CI 0.07–0.13; P<0.00001; χ^2 P=0.44; I^2=0%)
		Hospital admissions	Risk ratio 0.89 (95% CI 0.82–0.97; P=0.01; χ^2 P=0.58; I^2=0%)
		AECOPD	Risk ratio 0.80 (95% CI 0.69–0.92; P=0.002; χ^2 P<0.001; I^2=88%)
		HRQoL	SMD –0.13 (95% CI –0.16 to 0.10; P<0.00001; χ^2 P=0.67; I^2=0%)
		Treatment-related AEs	Risk ratio 0.99 (95% CI 0.97–1.01; P=0.34; χ^2 P=0.72; I^2=0%)
		Incidence of pneumonia	Risk ratio 1.07 (95% CI 0.93–1.23; P=0.36; χ^2 P=0.62; I^2=0%)

		All-cause mortality	Risk ratio 0.92 (95% CI 0.76–1.13; P=0.44; χ^2 P=0.99; I^2=0%)
		FEV_1 (L)	MD 0.08 (95% CI 0.06–0.09; P<0.00001; χ^2 P<0.0001; I^2=98%)
Rodrigo Int J Chron Obstruct Pulmon Dis 2017 [38]	LABA/LAMA vs LAMA or LABA/ICS (study duration of 12–64 weeks)	Trough FEV_1 (change from BL at 24–26 weeks, L)	Vs LAMA: MD 0.07 (95% CI 0.05–0.08; P<0.0001; I^2=56%)<br><br>Vs LABA/ICS: MD 0.06 (95% CI 0.00–0.12; P=0.04; I^2=90%)
		TDI focal score (change from BL at 24–26 weeks)	Vs LAMA: MD 0.29 (95% CI 0.12–0.46; P=0.0006; I^2=0%)
			Vs LABA/ICS: MD 0.33 (95% CI −0.28 to 0.95; P=0.29; I^2=0%)
		SGRQ (change from BL at 24–26 weeks)	Vs LAMA: MD −1.34 (95% CI −1.94 to −0.75; P<0.0001; I^2=0%)<br><br>Vs LABA/ICS: MD −1.13 (95% CI −1.78 to −0.48; P=0.0006; I^2=0%)
		Moderate/severe exacerbations	Vs LABA/ICS: risk ratio 0.82 (95% CI 0.75–0.91; P<0.0001; I^2=6%)
		Any AE	Vs LAMA: relative risk 1.00 (95% CI 0.98–1.02; P=0.95; I^2=0%)
			Vs LABA/ICS: relative risk 0.94 (95% CI 0.89–0.99; P=0.02; I^2=23%)
		Pneumonia	Vs LAMA: relative risk 1.04 (95% CI 0.78, 1.38; P=0.79; I^2=0%)
			Vs LABA/ICS: relative risk 0.59 (95% CI 0.43–0.81; P=0.001; I^2=0%)

Calzetta Eur Respir Rev 2017 [39]	LABA/LAMA vs LABA or LAMA	Trough FEV_1, SGRQ, TDI (3, 6 and 12 months)	LABA/LAMA always showed the highest probability of being the best therapy with regard to trough FEV_1, SGRQ and TDI at all time points (overall 98.67%) as confirmed by SUCRA (overall 99.28%); LAMA and LABA were ranked second and third
Calzetta Respir Med 2017 [41]	LABA/LAMA vs LABA or LAMA (study duration of 2–12 weeks)	Endurance time	LABA/LAMA: highest probability of being best therapy (100%), followed by LAMA (66%) and LABA (32%), as indicated by SUCRA analysis
		Inspiratory capacity	LABA/LAMA: highest probability of being best therapy (100%), followed by LAMA (64%) and LABA (36%), as indicated by SUCRA analysis
Aziz Int J Chron Obstruct Pulmon Dis 2018 [42]	LABA/LAMA vs SAMA, LAMA, LABA/ICS	FEV_1 (12–24 weeks)	LABA/LAMA ranked highest (SUCRA values ranging from 64.5% to 97.6%)
		TDI (12–24 weeks)	LABA/LAMA ranked highest (SUCRA values ranging from 53.5% to 97.8%)
		SGRQ (12–24 weeks)	LABA/LAMA ranked highest (SUCRA values ranging from 60.6% to 93.1%)
		AEs (12–24 weeks)	LABA/LAMA ranked highest (SUCRA values ranging from 33.7% to 83.9%)
Miravitlles Respir Res 2017 [56]	LABA/LAMA vs LAMA, LABA or LABA/ICS (study duration of 6–52 weeks)	FEV_1 (12 weeks)	Significantly higher trough FEV_1 vs LAMA (MD 0.06 [95% CI 0.04–0.07], I^2=33%) or LABA (MD 0.09 [95% CI 0.07–0.10], I^2= 0%); both P<0.00001
		TDI, change from BL	Significantly improved TDI vs LAMA (MD 0.44 [95% CI 0.22–0.65]; <0.0001; I^2=0%)
		SGRQ, change from BL	Significantly improved SGRQ vs LAMA (MD −1.56 [95% CI −2.41 to −0.71]; P=0.0003; I^2=0%)

		AEs	No significant difference vs LAMA or LABA (risk ratio 0.99 [95% CI 0.96–1.02]; P=0.57 I^2 = 0%)
Cazzola Eur Respir J 2018 [66]	LABA/LAMA vs LAMA, LABA or LABA/LAMA/ICS (treatment duration of 2–52 weeks)	Risk of AECOPD	Relative risk for LABA/LAMA/ICS vs LABA/LAMA: 0.86 (95%CI 0.75–0.99); I^2 (triple vs dual): 98%; P<0.01<br><br>SUCRA (highest to lowest): LABA/LAMA/ICS 0.99; LABA/LAMA 0.39; LAMA or LABA 0.12
		FEV_1 change from BL	MD for LABA/LAMA/ICS vs LABA/LAMA: 38.05 (95% CI 22.06–54.04); I^2 (triple vs dual): 45%; P=0.13<br><br>SUCRA (highest to lowest): LABA/LAMA/ICS 1.00; LABA/LAMA 0.50; LAMA or LABA 0.00
		Risk of pneumonia	Relative risk for LABA/LAMA/ICS vs LABA/LAMA: 1.31 (95% CI 0.97–1.76); I^2 (triple vs dual): 47%; P=0.11<br><br>SUCRA (highest to lowest): LABA/LAMA 0.73; LAMA or LABA 0.65; LABA/LAMA/ICS 0.12
		Overall weighted average of efficacy/safety	SUCRA (highest to lowest):<br>LABA/LAMA, 0.59;<br>LABA/LAMA/ICS 0.56;<br>LAMA or LABA 0.36
Calzetta Expert Rev Respir Med. 2021 [88]	LABA/LAMA/ICS vs LABA/LAMA or LABA/ICS (all fixed-dose combinations) (treatment duration of 24–52 weeks)	Moderate-to-severe AECOPD	SUCRA (highest to lowest): LABA/LAMA/ICS 0.99; LABA/ICS 0.51; LABA/LAMA 0.01
		FEV_1	SUCRA (highest to lowest): LABA/LAMA/ICS 0.98; LABA/LAMA 0.51; LABA/ICS 0.02

		Pneumonia	SUCRA (highest to lowest): LABA/LAMA 0.99; LABA/ICS 0.36; LABA/LAMA/ICS 0.15
Koarai Respir Investig 2022 [69]	LABA/LAMA vs LABA/LAMA/ICS	Exacerbations (24–52 weeks)	Significant decrease in number of exacerbations with LABA/LAMA/ICS vs LABA/LAMA (rate ratio, 0.56; 95% CI 0.38–0.85; P=0.006; I^2=16%)
		SGRQ (24–52 weeks)	No difference in SGRQ score change from BL between LABA/LAMA/ICS and LABA/LAMA treatment (MD –1.38; 95% CI –3.25 to 0.49; P=0.15; I^2=0%)
		Trough FEV_1 (24–52 weeks)	Significant increase in trough FEV_1 with LABA/LAMA/ICS vs LABA/LAMA treatment (MD 0.04; 95% CI 0.01–0.07; P=0.01; I^2=0%); however, difference was less than MCID of 0.05–0.10 L
		AEs (52 weeks)	No difference in total AEs between LABA/LAMA/ICS and LABA/LAMA (OR 1.07; 95% CI 0.68–1.67; P=0.77; I^2=0%)
		Pneumonia (52 weeks)	Significant increase in pneumonia events with LABA/LAMA/ICS vs LABA/LAMA (OR, 3.38; 95% CI 1.58–7.22; P=0.002; I^2=0%)
		Mortality (52 weeks)	No significant difference in mortality between LABA/LAMA/ICS and LABA/LAMA (OR, 2.91; 95% CI 0.47–18.05; P=0.25; I^2=0%)
Zheng The BMJ 2018 [70]	LABA/LAMA/ICS vs LABA/LAMA	Moderate to severe exacerbations (52 weeks)	Significantly reduced rate of moderate or severe exacerbations with LABA/LAMA/ICS vs LABA/LAMA (rate ratio 0.78; 95% CI 0.70–0.88; P=0; I^2=46.3)
		All-cause mortality (52 weeks)	No significant difference for all-cause mortality (risk ratio 0.77; 95% CI 0.58–1.03; P=0.07; I^2=0)

		Trough FEV_1 (L) (52 weeks)	Significant improvement in trough FEV_1 with LABA/LAMA/ICS vs LABA/LAMA (MD 0.04; 95% CI 0.02–0.07; P=0; I^2=27.1)
		AEs (52 weeks)	No significant difference for AEs (risk ratio 1.00; 95% CI 0.93–1.08; P= 0.98; I^2=58.3)
		Pneumonia events (52 weeks)	Significantly higher rate of pneumonia with LABA/LAMA/ICS vs LABA/LAMA (risk ratio 1.53; 95% CI 1.25–1.87; P=0; I^2=19.7)
		SGRQ (52 weeks)	Significant improvement in SGRQ score with LABA/LAMA/ICS vs LABA/LAMA (MD −1.81; 95% CI −2.57 to −1.04; P=0; I^2=0)
Koarai Respir Res 2021 [67]	LABA/LAMA/ICS vs LABA/LAMA	Exacerbations (24–52 weeks)	Significant decrease in exacerbations with LABA/LAMA/ICS vs LABA/LAMA (rate ratio 0.73; 95% CI 0.64–0.83; P<0.00001; I^2=78%)
		SGRQ (24–52 weeks)	Significant improvement in SGRQ score change from BL with LABA/LAMA/ICS (MD −1.71; 95% CI −2.27 to −0.92; P<0.00001; I^2=0%); however, difference was less than MCID of −4.0
		TDI (24–52 weeks)	Significant improvement in TDI score change from BL with LABA/LAMA/ICS (MD 0.33; 95% CI 0.18–0.48; P<0.00001; I^2=6%); however, difference was less than MCID of 1.0
		Trough FEV_1 (24–52 weeks)	Significant increase in trough FEV_1 with LABA/LAMA/ICS (MD 0.04; 95% CI 0.01–0.07; P=0.02; I^2=86%); however, difference was less than MCID of 0.05 to 0.10 L
		AEs (52 weeks)	No difference in total AEs between LABA/LAMA/ICS and LABA/LAMA (OR 1.03;

			95% CI 0.93–1.15; P=0.58; I^2=34%)
		Pneumonia events (52 weeks)	Significant increase in pneumonia events with LABA/LAMA/ICS (OR 1.52; 95% CI 1.16–2.00; P=0.003; I^2=32%)
		Mortality (52 weeks)	The incidence was small, but the frequency with LABA/LAMA/ICS was significantly lower (OR 0.66; 95% CI 0.50–0.87; P=0.003; I^2=0%)
Mammen Annals ATS 2020b [68]	LABA/LAMA/ICS vs LABA/LAMA	AECOPD (24–52 weeks)	Rate ratio 0.71 (95% CI 0.59–0.86; P=0.0003; I^2=78%)
		Pneumonia (24–52 weeks)	Risk ratio 1.41 (95% CI 1.05–1.89; P=0.02; I^2=28%)
		Dyspnea (14–52 weeks)	MD 0.09 (95% CI –0.01 to 0.19; P=0.09; I^2=0%)
		Mortality (24–52 weeks)	Risk ratio 0.73 (95% CI 0.53–1.02; P=0.06; I^2=0%)
		Treatment-emergent AEs (24–52 weeks)	Risk ratio 1.01 (95% CI 0.94–1.09); P=0.78; I^2=51%)
Lee PloS Med 2019 [74]	LABA/LAMA/ICS vs LABA/LAMA (study duration of 12–72 weeks)	Post- bronchodilator FEV_1% of predicted	SUCRA (highest to lowest): LABA/LAMA/ICS, 0.958; LABA/ICS, 0.777; LABA, 0.539; LABA/LAMA, 0.407; ICS, 0.340; Placebo, 0.244; LAMA, 0.235
		Total exacerbation in the past year (%)	SUCRA (highest to lowest): LABA/LAMA/ICS, 0.973; ICS, 0.697; LABA/ICS, 0.688; LABA/LAMA, 0.446; LABA, 0.359; LAMA, 0.203; placebo, 0.134
		mMRC scale	SUCRA (highest to lowest): LAMA, 0.775; LABA/LAMA/ICS, 0.668; LABA/LAMA, 0.525; ICS, 0.451; placebo, 0.430; LABA/ICS, 0.419; LABA, 0.233
		Reversibility (%)	SUCRA (highest to lowest): LABA/LAMA/ICS, 0.855;

			LABA/ICS, 0.726; LABA/LAMA, 0.553; LABA, 0.473; ICS, 0.466; LAMA, 0.262; placebo, 0.164

AE, adverse event; AECOPD, acute exacerbations of chronic obstructive pulmonary disease; BL, baseline; CI, confidence interval; COPD, chronic obstructive pulmonary disease; FEV_1, forced expiratory volume in 1 s; HR, hazard ratio; HRQoL, health-related quality of life; ICS, inhaled corticosteroid; LABA, long-acting β_2-agonist; LAMA, long-acting muscarinic antagonist; MCID, minimal clinically important difference; MD, mean difference; mMRC, modified Medical Research Council; N/A, not applicable; OR, odds ratio; RCT, randomized controlled trial; SAMA, short-acting muscarinic antagonist; SGRQ, St George's Respiratory Questionnaire; SMD, standardized MD; SUCRA, surface under the cumulative ranking curve; TDI, transitional dyspnea index.
